# Measuring health system resilience in a highly fragile nation during protracted conflict: South Sudan 2011–15

**DOI:** 10.1093/heapol/czz160

**Published:** 2019-12-26

**Authors:** Jackline Odhiambo, Caroline Jeffery, Richard Lako, Baburam Devkota, Joseph J Valadez

**Affiliations:** 1 Department of International Public Health, Liverpool School of Tropical Medicine, Pembroke Place, Liverpool L3 5QA, UK; 2 Directorate of Policy, Planning, Budgeting and Research, Ministry of Health, Juba, South Sudan

**Keywords:** Resilience, fragile and conflict-affected settings, FCAS, maternal and child health coverage, stress, complex adaptive systems, health system stress, South Sudan

## Abstract

Health systems resilience (HSR) is defined as the ability of a health system to continue providing normal services in response to a crisis, making it a critical concept for analysis of health systems in fragile and conflict-affected settings (FCAS). However, no consensus for this definition exists and even less about how to measure HSR. We examine three current HSR definitions (*maintaining function*, *improving function* and *achieving health system targets*) using real-time data from South Sudan to develop a data-driven understanding of resilience. We used 14 maternal, newborn and child health (MNCH) coverage indicators from household surveys in South Sudan collected at independence (2011) and following 2 years of protracted conflict (2015), to construct a resilience index (RI) for 9 of the former 10 states and nationally. We also assessed health system stress using conflict-related indicators and developed a stress index. We cross tabulated the two indices to assess the relationship of resilience and stress. For *maintaining function* for 80% of MNCH indicators, seven state health systems were resilient, compared with *improving function* for 50% of the indicators (two states were resilient). A*chieving the health system national target* of 50% coverage in half of the MNCH indicators displayed no resilience. MNCH coverage levels were low, with state averages ranging between 15% and 44%. Central Equatoria State displayed high resilience and high system stress. Lakes and Northern Bahr el Ghazal displayed high resilience and low stress. Jonglei and Upper Nile States had low resilience and high stress. This study is the first to investigate HSR definitions using a resilience metric and to simultaneously measure health system stress in FCAS. *Improving function* is the HSR definition detecting the greatest variation in the RI. HSR and health system stress are not consistently negatively associated. HSR is highly complex warranting more in-depth analyses in FCAS.

**Key Messages**

- Maternal, new-born and child health coverage indicators can be used to create a resilience index to measure health system resilience.
- In fragile and conflict-affected settings, resilience defined as a health system’s ability to improve its function identified the greatest variation in the resilience index in nine South Sudan State.
- Resilience and stress are not consistently negatively associated with each other, which supports the analysis of health systems as complex adaptive systems.
- Responsive governance, humanitarian aid and robust infrastructure may improve resilience.

## Introduction 

The concept of ‘building resilient health systems’ has been central to the development policy of multiple United Nations agencies and bilateral organizations since 2014 (DFID, 2011; IMF, 2015; Ziglio, 2017). Resilience is often presented as a management concept describing how systems respond to crises without disrupting their normal functions (Kruk *et al.*, 2015). However, several disciplines use the concept for different purposes which have obscured its meaning making it difficult to apply in health systems research. In ecology, resilience describes the ‘ability of ecological systems to absorb changes … and still persist’ (Holling, 1973), while in psychology it refers to the ability of individuals, households and communities to adapt positively to adversity (Olsson *et al.*, 2015). Resilience has also been applied to analyses of social-ecological and social systems, and more recently in health systems, with emphasis placed on fragile and conflict-affected settings (FCAS) (Zeid *et al.*, 2015; Spiegel, 2017).

However, there are two major intellectual gaps reducing the utility of the concept of ‘resilience’ when used to analyse health systems. For the purposes of this article, we refer to health systems resilience as HSR. Firstly, the definition of HSR lacks clarity and consensus. There are various HSR definitions highlighting different health system responses to stress as summarized in Table 1. These responses range from the ability of a system to resist, to absorb, to cope or to recover from multiple forms of stress. The definitions also include the ability of a system to evolve (to adapt or to transform) by introducing innovations following exposure to stress. These varied definitions do not make clear the distinction between whether ‘resilience’ is a system response to achieve a beneficial outcome or the outcome itself. Some of the definitions also assume that if a system is absorptive, adaptative or transformative, a resilience outcome will follow, which ignores the context and co-factors affecting the outcome.

**Table 1:** Summary of health system resilience definitions

Author	Resilience definition	Resilience process	Resilience outcome
Ammar *et al.* (2016)	The capacity of a health system to absorb internal or external shocks (e.g. prevent or contain disease outbreaks and maintain functional health institutions) while sustaining achievements.	Absorb	Sustain achievements Sustain or improve access Maintain function Long-term sustainability
	Resilience is the ability of a health system to sustain or improve access to healthcare services while ensuring long-term sustainability.		
Therrien *et al.* (2017)	The capacity/intrinsic ability of a social system (e.g. an organization, city or society) to proactively adapt to and recover from disturbances that are perceived within the system to fall outside the range of normal and expected disturbances/conditions so that it can sustain required operations.	Adapt Recover	Sustain required operations Recover from
Hanefeld *et al.* (2018)	HSR is about the system being able to adapt its functioning to absorb a shock and transform if necessary, to recover from disasters.	Absorb Adapt Transform	Recover from
Bayntun *et al.* (2012)	The capability of the public health and healthcare systems, communities and individuals to prevent, protect against, quickly respond to and recover from health emergencies, particularly those whose scale, timing or unpredictability threatens to overwhelm routine capabilities.	Prevent Protect against Respond to	Recover from
McKenzie *et al.* (2016)	Resilience is the capacity of health systems to deal with change, to adapt and transform and to maintain relevance when confronted by major disruptions	Adapt Transform	Maintain relevance
Blanchet *et al.* (2017)	The capacity of a [health system] to absorb, adapt [OR] transform when exposed to a shock such … armed conflict and still retain the same control over its structure and functions.	Absorb Adapt Transform	Control over structure and functions
Ager *et al.* (2015)	The ability… to manage change, by maintaining or transforming…standards in the face of shocks or stresses … without compromising … long-term prospects	Manage	Maintain standards Transform standards Long-term sustainability
Kruk *et al.* (2017)	The capacity of health actors, institutions and populations to prepare for and effectively respond to crises, maintain core functions when crisis hits and informed by lessons learnt during the crisis, re-organize if conditions require it.	Prepare for Respond to Learn Re-organize	Maintain function
Barasa *et al.* (2018)	A system’s ability to continue to meet its objectives in the face of challenges		Meet objectives

The second gap is that while all resilience definitions require exposure to stress (Table 1), none of the studies on HSR in FCAS, to our knowledge, has assessed the amount of stress in the study context, or the relationship between resilience and stress. Health system stress can take many forms; it can refer to both health-related stress such as disease outbreak, and non-health-related events such as military conflict, natural disasters or economic shocks. The relationship between resilience and stress is debated in the ecological literature pointing out a complex non-linear relationship between the two (Holling, 1973), whereas the sociological literature does not systematically investigate this relationship (Briguglio, 1995; Pratt *et al.*, 2004). Due to the implicit interaction of resilience and stress in humanitarian settings, investigations of this relationship may address questions such as: can health systems be resilient during acute and post-acute conflict settings?

These ambiguities stem from having few empirical assessments of HSR; a deficiency which may account for the lack of both validated HSR indicators and an evidence-based framework for measuring resilience in health systems (Thomas *et al.*, 2013). The few studies that have attempted to empirically describe HSR have used changes in population coverage of maternal, newborn and child health (MNCH) services, and maternal and child mortality rates (CMR; Ammar *et al.*, 2016; Qirbi and Ismail, 2017). These studies measured changes in five to six MNCH indicators, over a period of 1–24 years. However, none of the studies provided criteria for classifying a health system as resilient which left their conclusions subjective. Most of the studies assessed a single health system, whether national or regional; therefore, they have not been able to compare health systems in similar or different contexts. Despite these measurement and conceptual gaps, HSR is increasingly presented as a critical concept for making health systems programming decisions in FCAS and in forming related policies.

In this article, we attempted to address these deficiencies while clarifying the meaning of ‘resilience’ especially in FCAS. By doing so, we want to improve the utility of the concept for health systems strengthening. We carried out this research in the context of South Sudan, a highly fragile and conflict-affected country, and applied several definitions of resilience to learn which of them, if any, advances our understanding of how health systems perform in FCAS both nationally and at a sub-national level when affected by conflict stressors.

## Methods

### South Sudan

The 2018 Fragile States Index ranked South Sudan as the world’s most fragile country (Fund for Peace, 2018). After emerging from Africa’s longest civil war (1956–2005), the Republic of South Sudan attained independence in 2011, and shortly thereafter, in December 2013, it experienced more armed conflict. Conflict resolution has remained ineffective for many reasons such as political patronage, ethnic domination, elite power struggles and an international emphasis on state-building which supersedes building social cohesion and integration of ethnic and interest groups (Gerenge, 2016; Kane *et al.*, 2016).

Currently, at least half of the population in South Sudan lives below the World Bank’s poverty line and nearly three-quarters (73.5%) lack formal education. South Sudan has one of the world’s highest CMR (104 per 1000 live births) and maternal mortality ratios (730–789 maternal deaths/100 000 live births; Valadez *et al.*, 2015). These conditions are aggravated by nearly 1.97 million internally displaced people and 2.2 million refugees (UNHCR, 2018).

The Ministry of Health (MOH) of South Sudan established a national monitoring and evaluation system using household surveys to track the progress of health indicators. This survey measured coverage of MNCH services in each of the country’s former 10 states and counties. We used the data from these national surveys to investigate the HSR definitions. We also obtained information on conflict events routinely collected by the United Nations Office of Coordination of Humanitarian Affairs (UNOCHA). These latter data we used to measure health system stress. With both sources of data, we examined for the first time, HSR and its relationship with conflict-related health system stress. We did this to understand the utility of the HSR when evaluating progress of the health system of South Sudan which is a topic of interest to the MOH and bilateral and international donors.

### Household surveys

The MOH implemented two national cross-sectional household surveys using stratified random sampling during 2011 and 2015 in which the sampling domains were the 10 states; their counties (the administrative unit of the states) were the strata. This effort was undertaken to measure numerous MNCH indicators. Although details of the survey including its participants, sampling protocols and results can be found elsewhere (Valadez *et al.*, 2015; Republic of South Sudan, 2018), we briefly summarize them here. The MOH used two-stage sampling in each county. Firstly, villages were sampled in each state county with probability proportional to size. In each village, trained data collectors used segmentation sampling (Turner *et al.*, 1996; Davis and Valadez, 2014) to randomly select households for interview. One person in the household was randomly selected using a random number table when more than one was eligible. Study participants included women of reproductive age (15–49 years), and mothers of children 0–11, 12–23 and 6–59 months, and those with children 0–59 months with diarrhoea, suspected pneumonia or malaria in the last 2 weeks. Sampling continued in each village until one person in each cohort was selected. Each sampling unit had its own independent sample, and the total sample collected in 2011 was (1475 × 7 cohorts) 10 325, and 9443 (1349 × 7 cohorts) in 2015.

Data collectors were State MOH health workers associated with the monitoring and evaluation units, who were trained and supervised by technical advisors from the Liverpool School of Tropical Medicine to use the study protocols and pretested standardized questionnaires. Questions were asked in the local language or in Arabic. County level data were weighted by their population sizes and aggregated to produce state and national level coverage estimates with 95% confidence intervals. For this study, we used state and national weighted coverage estimates for 14 MNCH indicators to measure resilience outcome (Table 2), calculated using Stata-v14 (Statistical Software, College Station, TX; StataCorp LP, 2011), Excel-v2013 and R-v3.2.3.

**Table 2:** MNCH coverage indicators in South Sudan

Indicator domain	Indicator short code	Indicator
Maternal and newborn health	Contraceptive prevalence	Proportion of women 15–49 years and not pregnant using any modern family planning method at the time of the survey.
	4+ ANC visits	Proportion of mothers of children 0–11 months who had at least four ANC visit during their last pregnancy.
	2+ tetanus toxoid vaccination during last pregnancy	Proportion of mothers of children 0–11 months who received two or more doses of tetanus toxoid during their last pregnancy or who had life-time immunity.
	Malaria prophylaxis: IPT2	Proportion of mothers of children 0–11 months who received two or more doses of SP Fansidar/Intermittent Prevention therapy (IPT) for malaria during their last pregnancy.
	Skilled birth attendance	Proportion of mothers of children 0–11 months who delivered in the presence of skilled health personnel during their last pregnancy.
	1+ Postnatal care visit	Proportion of mothers of children 0–1 months who had at least one postnatal care visit within 6 weeks of delivery with a skilled health professional.
	Slept under LLIN/ITN night of survey	Proportion of mothers of children 0–59 months who slept under an LLIN/ITN the night preceding the survey.
Child health	Vitamin A supplementation	Proportion of children 6–59 months who received Vitamin A supplement in the last 6 months
	DPT3 vaccination	Proportion of children 12–23 months who received DPT3 vaccine before first birthday (card and recall).
	Full vaccination	Proportion of children 12–23 months who are fully vaccinated (BCG, DPT3, OPV3 and measles) before their first birthday (card and recall).
	U5 slept under LLIN/ITN night of survey	Proportion of children 0–59 months who slept under an LLIN/ ITN the night preceding the survey.
	U5 diarrhoea treatment with ORS	Proportion of children 0–59 months with diarrhoea in the 2 weeks before the survey who were treated with ORS.
	U5 ARI treatment with appropriate antibiotics	Proportion of children 0–59 months with cough and fast/difficult breathing in the 2 weeks before the survey who were treated with an appropriate antibiotic (as per national guidelines).
	U5 fever treatment with appropriate anti-malarial	Proportion of children 0–59 months with fever in the last 2 weeks who were treated with an appropriate anti-malarial (as per national guidelines).

MNCH, maternal, new-born and child health; ANC, antenatal care; IPT2, intermittent prevention therapy; DPT3, diphtheria-pertussis-tetanus; BCG, Bacillus Calmette–Guerin; OPV3, oral polio vaccine; LLIN, long lasting insecticide-treated bednet; ITN, insecticide-treated bednet; U5, under-five; ORS, oral rehydration solution.

### Conflict dataset

We obtained the conflict dataset from UNOCHA in South Sudan who captured states-level conflict data from media and intelligence reports. We used three conflict indicators measured during 2011–15. We used total reported conflict incidents to measure exposure to conflict. We defined conflict incidents as any conflict event involving military forces, police forces, rebel forces, ethnic militia or civilian protests and including activities such as bombing, air attacks, raids, shootings and cattle raiding. The total reported conflict-related fatalities was a proxy measure of the severity of conflict which affects access to healthcare due to limited movements and reduces availability of healthcare due to destruction of health facilities. We used the total number of internally displaced persons (IDPs) arriving into the state (these data are for 2013–15 as they were not available for 2011) to measure the burden of care in the host state health system either due to sharing health resources with disbursed IDPs or by transferring health resources, such as healthcare providers, to IDP camps.

### Testing resilience definitions

HSR concerns the systemic response to crisis events; for this reason, we tested for difference in coverage with several MNCH interventions and services during 2011 and 2015 using a two-tailed two sample test for binomial proportions with a normal approximation, including a continuity correction to account for the binomial distribution (Rosner, 2015, pp. 373–386). However, for cases where the expected cell frequencies were less than five the two-tailed test would violate the assumptions for normal approximation; we, therefore, used a Yates-corrected chi-square test to prevent overestimation of *P*-values for small data. We tested for differences at *P* ≤ 0.05.

We used thematic analysis to synthesize the HSR literature to arrive at three definitions of resilience: *maintaining function*, *improving function* and *achieving the health system’s goal* (Table 3). We queried each of the definitions with sensitivity analyses. Firstly, we defined the dominant definition of *maintaining function* as at least 80% of the 14 MNCH services *maintained or improved* their indicator values during the 2011–15 period. In sensitivity analysis, we also tested this definition for 100% and 50% of the indicators improving. Secondly, we set the definition of *improving function* at 50% of the MNCH services *improved* and also tested it for 80%, 40% and 30% of the indicators. Lastly, for the definition of *achieving a health system coverage target*, we defined resilience as at least half of the MNCH indicators achieving a 50% coverage target by 2015 since this was the coverage target established by the MOH in both 2011 and 2015 (Valadez *et al.*, 2015). We also tested coverage targets of 40% and 30% in sensitivity analyses. We used significance tests for *maintaining* and *improving function* and spreadsheets to depict achievement of the health system target*.* Our analysis included data from only nine states as Unity State was under rebel control in 2015 preventing data collection.

**Table 3:** Resilience definitions based on resilience outcome

HSR working definitions	Ability of a health system to maintain/improve its functions or meet health system objectives despite crisis	
Primary analysis	Definition with sensitivity analyses	Indicator scoring
Definition 1: Resilience as *maintaining health system function*	At least *X*% of the MNCH indicators were maintained (did not change statistically significantly or improved statistically significantly) between 2011 and 2015	Score = 1 if state had a statistically significant improvement or non-statistically significant change in MNCH coverage
	*X*_1_ = 100%, *X*_2_ = 80%, *X*_3_ = 50%	Score = 0 if state had a statistically significant decline in MNCH coverage
Definition 2: Resilience as *improving health system function*	At least *X*% of the MNCH indicators improved statistically significantly between 2011 and 2015	Score = 1 if state had a statistically significant improvement in MNCH coverage
	*X*_1_ = 80%, *X*_2_ = 50%, *X*_3_ = 40%, *X*_4_ = 30%	Score = 0 if state had non-statistically significant change OR had a statistically significant decline in MNCH coverage
Definition 3: Resilience as *achieving health system’s targets*	At least half of the MNCH indicators met the health system coverage goal of *Y*% in both years or in 2015 only	Score = 1 if indicator coverage is ≥50% in both 2011 and 2015 or ≥50% in 2015 but <50% in 2011 (this would show improvement)
	*Y*_1_ = 50%, *Y*_2_ = 40%, *Y*_3_ = 30%	Score=0 if indicator coverage is <50% in both 2011 and 2015 or is ≥50% in 2011 but <50% in 2015 (this would show decline)

HSR, health system resilience; MNCH, maternal, newborn and child health.

### Resilience and stress indices

To compare HSR and the amount of stress placed on the health system, we constructed a resilience index (RI) and health system stress index (SI) by adapting Briguglio’s formula for calculating economic vulnerability (Briguglio, 1995). When testing the three resilience definitions, we coded resilience as a binary yes/no outcome. However, to build the RI, we treated resilience as a continuum ranging from high to low. To generate RI, we first summed the per cent coverage difference between 2011 and 2015 for all indicators at state and national levels. This produced the total percentage difference (Total D%) which we used in the following formula to calculate a RI for each state and the national health system.

$${\mathrm{RI}}_{\mathrm{per}\mathrm{system}}=\frac{\sum_{1}^{14}{D\%}_{\mathrm{per}\mathrm{system}}-\min\sum_{1}^{14}{D\%}_{\mathrm{across}\mathrm{systems}}\;}{\max\sum_{1}^{14}\;{D\%}_{\mathrm{across}\mathrm{systems}}-\min\sum_{1}^{14}{D\%}_{\mathrm{across}\mathrm{systems}}\;}$$

*Note: per system refers to a specific state (e.g. Central Equatoria) or national (South Sudan) health system being compared with the least performing state health system (min Total D% across systems) and the best performing state health system (max Total D% across systems)* *.*

The index ranged between one (most resilient) and zero (least resilient). We also conducted sub-group analyses for the RI, testing for state and national performance for maternal and child indicators separately.

For health system stress, we first mapped each state’s total conflict fatalities (<2000 fatalities vs ≥2000 fatalities) and total number of IDP arrivals (<200 000 vs ≥200 000) to visualize the distribution of stress. Because stress variables (conflict incidents, fatalities and numbers of IDPs) were in different units, we developed a SI for each variable individually. For each of the three variables, we first calculated the annual number of conflict incidents, fatalities and number of IDPs per state and at the national level. We then used the annual number for each variable, e.g. IDPs, to generate a SI for IDPs per state and nationally using the following formula:

$${\mathrm{SI}}_{\mathrm{for}\mathrm{IDPs}\mathrm{per}\mathrm{system}}=\frac{{\mathrm{Annual}\#\mathrm{of}\mathrm{IDPs}}_{\mathrm{per}\mathrm{system}}-\min{\mathrm{Annual}\#\mathrm{of}\mathrm{IDPs}}_{\mathrm{across}\mathrm{systems}}}{{\max\mathrm{Annual}\#\mathrm{of}\mathrm{IDPs}}_{\mathrm{across}\mathrm{systems}}-\min\mathrm{Annual}\#\mathrm{of}{\mathrm{IDPs}}_{\mathrm{across}\mathrm{systems}}}$$

*Note: per system refers to a specific state (e.g. Central Equatoria) or national (South Sudan) health system being compared with the state health system with the least amount of stress value such as least annual number of IDPs (min amount of stress value across systems) and the state health system with the highest amount of stress value e.g. highest annual number of IDPs (max amount of stress value across systems)* *.*

We repeated this formula for the annual number of conflict incidents and conflict fatalities and generated three health system stress indices for each state. We then took the arithmetic mean of the three SI to generate an overall SI for each state and the national health system, with values ranging between one (highest stress—most affected by the three variables combined) and zero (least stress). We weighed all three variables equally to avoid overemphasizing-related variables (conflict incidents increase likelihoods of fatalities and IDPs; Brooks *et al.*, 2005), and avoid predicting an outcome by weighting one variable greater than the other which is contrary to resilience theory’s assumptions of unpredictable outcomes (Holling, 1973). States receiving more IDPs or with more fatalities experience greater pressure for services either due to an increased population in need or by having fewer functional services and facilities, respectively. Finally, we cross tabulated RI and SI using Briguglio’s vulnerability and resilience framework. We also conducted sub-group analyses of maternal and child services to identify indicator domains displaying differential levels of resilience.

### Ethics

The household surveys were reviewed and approved by the LSTM Research Ethics Committee and the Ethics Review Committee of the Ministry of Health of South Sudan. UNOCHA’s conflict data are secondary anonymized datasets, this study received ethics exemption from LSTM Research Ethics Committee.

## Results

### HSR definitions

The three definitions for HSR produced different results. *Definition 1*: For *maintaining function for ≥80%* of the indicators, 7 of 10 health systems assessed displayed resilience (Table 4) as ≥80% of the MNCH indicators were either maintained or improved in six states and for the nation as a whole. None of the health systems maintained 100% of the MNCH services. But, all the health systems displayed resilience for *maintaining 50%* of the MNCH indicators.

**Table 4:** Test results for HSR definitions in South Sudan using 14 MNCH indicators at state and national health system levels

Resilience definitions	Central Equatoria	Eastern Equatoria	Jonglei	Lakes	Northern Bahr el Ghazal	Upper Nile	Warrap	Western Bahr el Ghazal	Western Equatoria	National
Maintaining function										
≥80% of the indicators maintained										
100% of the indicators maintained										
≥50% of the indicators maintained										
Improving function										
≥50% of the indicators improved										
≥80% of the indicators improved										
≥40% of the indicators improved										
≥30% of the indicators improved										
Achieving health system’s targets										
At least half of the indicators met 50% health coverage goal										
At least half of the indicators met 40% health coverage goal										
At least half of the indicators met 30% health coverage goal										

Maintaining function: *X*% of indicators did not change significantly or improved significantly between 2011 and 2015; improving function: X% of indicators statistically significantly improved between 2011 and 2015; achieving health system’s targets: X% of indicators met the health system coverage target of Y% in both 2011 and 2015 or unmet in 2011 but met in 2015:

States with positive results (resilient) States with negative results (not resilient).

HSR, health system resilience; MNCH, maternal, newborn and child health.

*Definition 2*: For *improving function in ≥50%* of the indicators, two health systems (Central Equatoria and the nation as a whole) produced positive results (Table 4). None of the health systems had positive results for *improvement in* ≥80% of the MNCH indicators, but 7 of 10 health systems showed positive results for *improvement in ≥40%* of the MNCH services.

*Definition 3*: For *achieving health system coverage goal of 50% in half of the indicators*, no health system had a positive result (Table 4). Only Central Equatoria displayed resilience when the coverage target reduced to 40%. At a health system coverage target of 30%, Western Bahr el Ghazal and Western Equatoria also displayed resilience. The coverage for most of the 14 indicators was below 50% in both 2011 and 2015 in all states (Supplementary Table S1). Only Central Equatoria had more than 3 of 14 indicators improving to at least 50% coverage by 2015.

### Resilience and stress indices

**Figure 1:**
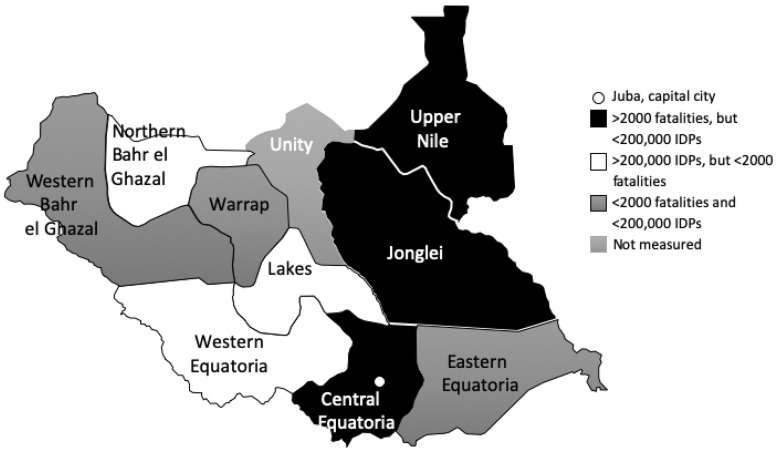
Geographic distribution of health system stress (conflict-related stress) in South Sudan between 2011 and 2015.

Central Equatoria, Jonglei and Upper Nile had the largest number of conflict-related fatalities (Figure 1). Lakes, Northern Bahr el Ghazal and Western Equatoria received the most IDPs.

**Figure 2:**
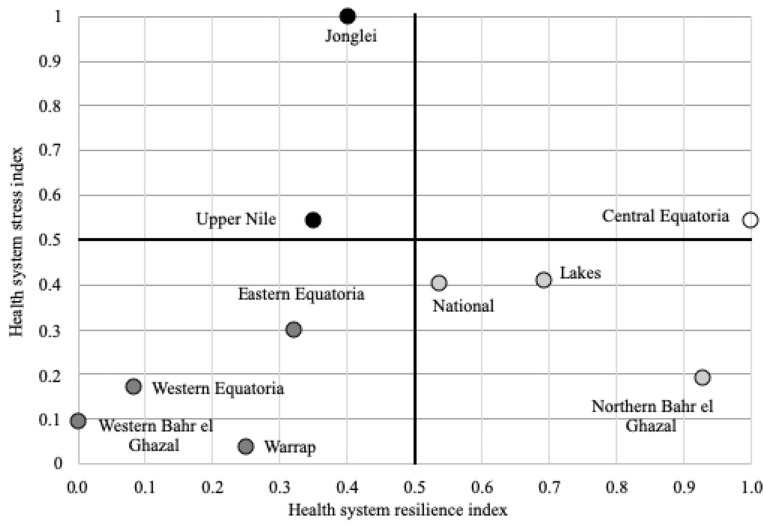
Health system resilience and stress matrix at state and national levels in South Sudan. (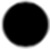) Low resilience and high stress, (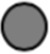) Low resilience and low stress, (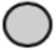) High resilience and low stress, (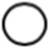) High resilience and high stress.

Considering all MNCH indicators, the most resilient state was Central Equatoria (RI = 1.000), followed by Northern Bahr el Ghazal (RI = 0.927), Lakes (RI = 0.692) and Jonglei (RI = 0.402; Figure 2). The least resilient states were Western Bahr el Ghazal (RI = 0.000), Western Equatoria (RI = 0.083), Warrap (RI = 0.250), Eastern Equatoria (RI = 0.321) and Upper Nile (RI = 0.351). The national health system ranked fourth with an RI of 0.537.

The state with the highest amount of health system stress was Jonglei (SI = 0.999), followed by Upper Nile (SI = 0.543), Central Equatoria (SI = 0.542) and Lakes (SI = 0.408; Figure 2). The amount of stress in these four states was at least twice the magnitude of the stress in the four states rated as having low stress. The states with lowest stress were Warrap (SI = 0.038), followed by Western Bahr el Ghazal (SI = 0.093), Western Equatoria (SI = 0.171) and Northern Bahr el Ghazal (SI = 0.191). The national health system ranked fifth in the health system stress index (SI = 0.401).

Using the Briguglio’s resilience and vulnerability framework, Central Equatoria displayed high resilience and high health system stress (Figure 2). Lakes and Northern Bahr el Ghazal showed high resilience and low stress. Jonglei and Upper Nile had low resilience and high stress. The rest of the states (Eastern Equatoria, Western Equatoria, Warrap and Western Bahr el Ghazal) displayed low resilience and low stress. Compared with its states, the national health system was highly resilient and experienced low health system stress.

**Figure 3:**
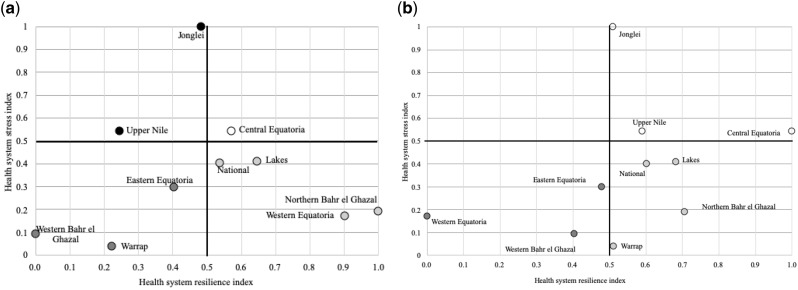
(a) Health system resilience and stress matrix at state and national levels in South Sudan focused on maternal and newborn health. (b) Health system resilience and stress matrix at state and national levels in South Sudan focused on child health. (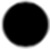) Low resilience and high stress, (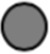) Low resilience and low stress, (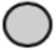) High resilience and low stress, (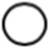) High resilience and high stress.

To understand whether maternal and child services display differing amounts of resilience measurement, we assessed them separately. Four of the seven indicators that improved in at least five of the health systems were child health indicators: DPT3 and full vaccination (eight health systems each), under-5 years malaria and diarrhoea treatment (each in six states; Supplementary Table S2). The only maternal indicators improving were coverage of pregnant women with two doses of tetanus toxoid vaccine, which improved significantly in all states. Maternal postnatal care visit and at least two doses of malaria prevention therapy also improved in six and five health systems, respectively. In the sub-group analyses, the RI results of maternal services remained similar to that of all-MNCH indicator analysis, except for Western Equatoria, which had low resilience for all MNCH indicators but high resilience for maternal indicators alone (Figure 3a). For child indicators, the RI results were different from that of all-MNCH indicator analysis. Seven of the 10 health systems including Jonglei, Upper Nile and Warrap displayed high resilience compared with four health systems for all MCNH indicators (Figure 3b).

## Discussion

### HSR definitions

This is the first study to examine HSR definitions using real-world data in a highly fragile-country setting. By so doing, it contributes to the development of an HSR evidence base. The definition of *maintaining function* for which ≥80% of indicators either did not lose value or increased coverage, can be applied to most of the South Sudanese state health systems. This definition was not useful for understanding differences in health system performance in the various cultural settings, or for understanding the processes for strengthening health systems. Coverage rates for most services in 2011 were very low and several of the detected increases in 2015 were not statistically significant. This condition highlights the difficulty of applying the concept of resilience in a fragile setting. As many of the indicators were already very low, the data are possibly revealing a floor effect—a situation that could not deteriorate further. However, it may also suggest that in fragile settings, if a nation is still able to maintain its coverage even at low levels, it is demonstrating resilience. For example, Jonglei and Upper Nile had acute health system stress but the status of their health indicators was maintained. Central Equatoria had high resilience but less acute protracted stress. Nevertheless, maintaining low coverage has negative implications for maternal and child survival; hence, even if South Sudan is considered as having a resilient health system, it is far from being in a satisfactory condition. Therefore, the definition of *maintaining function* may be less useful for analyses of health systems in highly fragile settings.

The HSR definition of *improving function* may be more appropriate for highly FCAS. Two of the 10 health systems (Central Equatoria and the nation as a whole) displayed high resilience by this criterion when 50% of indicator improvement was used as the standard. However, for a new nation and one which is still fragile and in conflict, improvement in >50% of the indicators or achieving a coverage target of 50% might be setting the threshold level too high (Valadez *et al.*, 2015; Kruk *et al.*, 2017). Achieving a health system target might be a less useful resilience definition as none of the state health systems achieved the 50% coverage target. Using a resilience definition as reaching a coverage target, reduces our ability to compare the performance of different states and draw lessons from them.

This study suggests that the definition of *improvement i*s more appropriate for a FCAS with very low initial coverage, as it revealed the variation in performance across the states, and formalizes the meaning of ‘strengthening’ in the concept of health systems strengthening. The challenging question concerns how to set the threshold of the percentage of indicators to detect improvement or even how to set a health system coverage target in the first place. These considerations are essential for measuring a country’s health system resilience. A principled solution is needed for HSR to be meaningful (Ioannidis, 2005). Future research needs to consider this point systematically.

### The relationship of resilience and system stress in health systems

Central Equatoria exhibited high stress and high resilience. Other studies in South Sudan show Central Equatoria leading in MNCH coverage despite major inter-tribal conflict (Valadez *et al.*, 2015). This counter intuitive result may be due to the national capital, Juba, being located in Central Equatoria which has a large amount of internal control and security by the military, resulting in better responsiveness and access to health resources than in other states. More than 40% of the population in Central Equatoria, on average, live within 5 km of a functional health facility (Macharia *et al.*, 2017). At least half of the doctors, midwives and laboratory technicians, and a third of the nurses and clinical officers in South Sudan work in Central Equatoria (Ministry of Health, 2012). Central Equatoria also hosts the majority of the government’s humanitarian and development partners which benefit from the large presence of security forces and better communication infrastructure than other parts of the country. Thus, Central Equatoria’s higher level of resilience may be due to it relatively better governance, which has been shown in other FCAS to be an important factor (Blanchet *et al.*, 2017). We should also note that the conflict in Central Equatoria was less protracted and the military used less high-grade military equipment as compared with Jonglei and Upper Nile. The duration of conflict events may also be an important factor to understand resilience. This variable we have yet to consider.

Despite having the highest number of IDPs, Lakes and Northern Bahr el Ghazal health systems displayed low health system stress and high resilience. The high number of IDPs in these states may have attracted more relief efforts than other states, and possibly heightened local leadership capacity and the effective co-ordination of relief plans by the multiple agencies, which have been effective elsewhere (WHO, 2014; Hanefeld *et al.*, 2018). The merging of donor resources in a Health Pool Fund (HPF) increased the diversity and the amount of primary care services, both of which should increase resilience (Kruk *et al.*, 2017). However, HPF works in Warrap, Western Bahr el Ghazal, Eastern Equatoria, Lakes and Northern Bahr-el Ghazal (Integrity, 2018) and did not have an apparent uniformly positive impact on resilience. HPF is a consortium and contracts implementing organizations, which are NGOs, to deliver services in different parts of a State; the organizations working in one State sometimes work in other States. As a result, the conditions to which they are exposed in one location may be different to conditions in another one. For example, areas of Lakes, Western Bahr el Ghazal and Northern Bahr el Ghazal have a more developed road network, which improved the logistics of service delivery in those locations (Macharia *et al.*, 2017) and facilitated international relief efforts.

Similar to other studies, which found that the population of Jonglei and Upper Nile had low access to health services and low health facility performance, in this current study, the two states displayed a high stress level and low resilience (Macharia *et al.*, 2017). Both states are in oil rich areas and have endured the brunt of the protracted conflict both before South Sudan’s independence and after the recurrence of violence in December 2013. These conditions weakened these states’ capacity to provide health services (Valadez *et al.*, 2011). A quarter of health facilities in these states is non-functional and only <10% of the population live within 1 h of a functional health facility (Macharia *et al.*, 2017). Because of the high insecurity, public and civil society health implementing partners experience many challenges. In addition to armed conflict and the capturing of materials by armed forces, they are affected by a high attrition of health workers and a weakened state governance capacity to implement the government’s health policies (Ministry of Health, 2012). The World Bank (2015) alone funds service delivery in both states; and states maintained service coverage at a pre-December 2013 level.

Warrap, Eastern Equatoria, Western Bahr el Ghazal and Western Equatoria health systems had a low amount of stress and low resilience. While stress due to conflict may have been low in these states, stress due to other factors, such as the impact of the economic downturn in South Sudan, inadequate health workers coupled with strikes and attrition, and limited governance capacity, may have contributed to the low resilience. Several counties in these states are remote with poor infrastructure where <25% of the population live within 1-h walk of a functional health facility (Macharia *et al.*, 2017). Other studies in South Sudan also show rural–urban disparities in MNCH coverage of which these states are good examples of rural settings (Mugo *et al.*, 2015). In these remote regions, at least 40% of health facilities are non-functional due to lack of human resources (WHO, 2014). Little infrastructure to provide health services and fewer health system resources retards measurable resilience.

Overall, state health systems in South Sudan experienced different types and amounts of vulnerabilities, but their vulnerabilities are interrelated (Keohane and Nye, 1987). For example, IDPs crossed state boundaries from high-conflict to low-conflict states. Some states scored low on the health systems SI due to experiencing less conflict, but they might experience other types of stress not measured in our study such as geographical inaccessibility. Health policies aimed at strengthening the health system need to be attuned to each state’s specific vulnerabilities, and state actions need improved co-ordination between the state ministries of health. These two actions are essential for improving health system resilience (Blanchet *et al.*, 2017). For example, the HPF operates in five states. Lessons on resource co-ordination among the HPF consortium members in the more resilient states could facilitate identification of policies and practices to mitigate the amount of stress and improve resilience in the other currently less resilient states (Brooks *et al.*, 2005).

This first analysis of HSR and its relationship with health system stress for FCAS indicated that health systems are complex adaptive systems (CAS; Lansing, 2003), as several different outcomes resulted with different levels of improvement occurring in different states. The 14 MNCH indicators revealed, as observed in other FCAS literature, some health system domains (child health) showed more resilience than others (maternal health) indicating that while health systems evolve in contexts of violence, they may do so selectively by prioritizing some services. Health system actors in South Sudan, including caregivers, may have selected child health as the domain in which to first build resilience. It might also be that health systems have different rates for building resilience for different service domains. Much about the process of HSR is yet to be well understood.

Contrary to currently held assumptions that increased stress reduces resilience, system stress was not necessarily negatively associated with resilience (Therrien *et al.*, 2017). This limited association between resilience and stress in our study might be due to the limitations of our SI, which measured only conflict-related stress indicators in an extremely fragile nation. However, the features of CAS, such as diversity of actors, redundancy of services, interdependence and adaptation may explain some of our results, but theory in the absence of data leaves much to speculation, which is not beneficial to policymakers (Kruk *et al.*, 2017). CAS theory is increasingly being used to understand resilience and health systems; however, more evidence-based research is needed to validate HSR frameworks.

### Study limitations

This study assessed health system outcomes to measure resilience, but it did not assess the processes of achieving resilience such as a state’s prior history of dealing with stress, the amount of existing social capital, and available health resources such as the number of staff and health facilities. Including these dimensions in future research may increase insight about factors associated with improving HSR in FCAS. Secondly, we only measured coverage indicators to assess resilience; subsequent studies should include the quality of clinical care as well (Valadez *et al.*, 2015). Furthermore, we merged 14 coverage indicators to generate a RI. Measuring each indicator separately with one resilience definition will improve understanding of the variability of response in different areas of the health system, e.g. various child care services compared with maternal care services or different conditions of stress in the health system.

Statistical significance tests for HSR definitions assumed resilience was a binary outcome rather than a continuum; we, therefore, used the per cent difference in coverage to build a RI with a large total per cent difference ranking high in resilience. Although there is precedent for this approach, future research should explore resilience as a continuum. The national health system ranked as having high resilience when using significance tests compared with most states; this is because it had a large sample size due to aggregating data from nine states, resulting in more power to detect statistical difference.

This study used three measures of health system stress related to conflict; however, additional measures of stress should be considered such as the economic shocks in South Sudan, inadequacy and attrition of healthcare workers, as well as strikes, weak governance capacity, social capital, ethnic diversity, population density, road density, wealth index, duration of conflicts or possibly dietary diversity (Pratt *et al.*, 2004; Sprague *et al.*, 2016). The conflict incidents were captured through radio and print media as well as intelligence reports, but they may have been under-reported in the most remote areas of South Sudan. We excluded Unity state from the analysis as it was under rebel control and not accessible in 2015. Including it would have provided additional comparisons with Jonglei and Upper Nile with similarly high conflict, thereby reducing state selection bias. Finally, we did not weight the health system stress indicators differently as they were interrelated and no criteria were established for doing so. Future research should consider weighting the indicators in the SI.

## Conclusion

This, the first study testing HSR definitions, used real-world data to measure HSR and cross tabulated it with the amount of stress evident in a FCAS. Defining HSR as the ability of a health system to statistically *improve* services, despite protracted crisis, was more appropriate in a FCAS than defining HSR as the ability to *maintain function*. The floor effect of the indicator values rendered *maintenance* a less useful concept. Resilience and health system stress were not necessarily negatively associated. Other mitigating factors exist. Improved local governance, access to health resources and robust humanitarian aid can improve health system resilience in the presence of high levels of stress. This conclusion may not extend to areas with acute stress. In those settings, *maintenance* may be the preferred definition to use, as a shorter-term HSR strategy. Our conclusions demonstrate the importance of empirical assessment of resilience and suggest directions for future HSR research. We should improve the measurement of resilience and stress indices through complex models containing additional population, military, political and geographical variables. We should also continue to track progress of FCAS to more develop a more robust theory of resilience for the future.

## Supplementary Material

czz160_Supplementary_Data
