# Supplementary material for: Measuring health system resilience in a highly fragile nation during protracted conflict: South Sudan 2011–15
Source: Health Policy Plan. 2019 Dec 26;35(3):313–22. doi: 10.1093/heapol/czz160 (PMC7152724; doi:10.1093/heapol/czz160)
Supplement: czz160_Supplementary_Data [file czz160_supplementary_data.doc]

**Supplementary Table S1. Coverage levels for MNCH indicators in South Sudan in 2011 and 2015**

| **States** | **Central Equatoria** | | **Eastern Equatoria** | | **Jonglei** | | **Lakes** | | **Northern Bahr el Ghazal** | | **Upper Nile** | | **Warrap** | | **Western Bahr el Ghazal** | | **Western Equatoria** | | **National** | |
| --- | --- | --- | --- | --- | --- | --- | --- | --- | --- | --- | --- | --- | --- | --- | --- | --- | --- | --- | --- | --- |
| **Year** | 2011 | 2015 | 2011 | 2015 | 2011 | 2015 | 2011 | 2015 | 2011 | 2015 | 2011 | 2015 | 2011 | 2015 | 2011 | 2015 | 2011 | 2015 | 2011 | 2015 |
| Contraceptive prevalence | 9.9 | 10.6 | 5.9 | 4.6 | 3.6 | 0.3 | 5.4 | 2.3 | 3.3 | 3.3 | 4.7 | 3.0 | 2.5 | 1.2 | 10.2 | 2.3 | 20.3 | 17.0 | 6.5 | 5.0 |
| 4+ ANC visits | 40.1 | 39.7 | 11.1 | 18.6 | 4.5 | 11.0 | 15.1 | 18.3 | 24.9 | 27.8 | 17.4 | 22.4 | 11.4 | 9.6 | 36.8 | 38.6 | 38.6 | 28.8 | 20.0 | 22.6 |
| 2+ maternal tetanus toxoid vaccination | 30.2 | 73.3 | 5.4 | 37.8 | 4.2 | 39.8 | 0.0 | 50.0 | 9.5 | 58.9 | 7.7 | 37.7 | 5.4 | 40.0 | 18.3 | 58.1 | 14.2 | 57.0 | 10.4 | 50.1 |
| 2+ maternal IPT2 doses | 36.9 | 42.0 | 12.2 | 21.8 | 17.1 | 30.6 | 19.8 | 33.8 | 35.1 | 42.8 | 16.5 | 34.9 | 25.0 | 23.3 | 36.2 | 37.2 | 30.9 | 35.9 | 23.7 | 32.9 |
| Skilled birth attendance | 36.8 | 49.0 | 15.5 | 19.1 | 7.2 | 13.6 | 9.9 | 25.3 | 16.8 | 22.2 | 31.2 | 25.4 | 13.2 | 13.2 | 55.5 | 59.7 | 34.7 | 43.1 | 22.1 | 27.5 |
| 1+ postnatal care visit | 12.0 | 22.0 | 7.0 | 15.8 | 2.3 | 8.9 | 9.5 | 7.1 | 6.7 | 26.5 | 15.7 | 12.0 | 8.6 | 14.1 | 18.1 | 5.3 | 5.7 | 31.4 | 9.0 | 16.7 |
| DPT3 vaccination | 33.2 | 67.0 | 17.8 | 37.6 | 22.7 | 23.4 | 9.6 | 21.8 | 9.4 | 19.1 | 20.8 | 24.6 | 12.0 | 20.2 | 32.7 | 47.9 | 28.0 | 40.9 | 19.5 | 33.8 |
| Full vaccination | 15.6 | 48.0 | 11.6 | 23.2 | 9.8 | 10.0 | 0.0 | 15.3 | 6.5 | 11.3 | 4.7 | 17.7 | 1.5 | 7.0 | 12.9 | 41.6 | 5.4 | 16.9 | 7.6 | 20.4 |
| U5 slept under LLIN or ITN | 46.8 | 42.0 | 23.9 | 4.7 | 37.0 | 22.5 | 27.8 | 23.7 | 8.6 | 11.3 | 34.2 | 31.1 | 24.6 | 11.6 | 9.2 | 0.7 | 31.6 | 7.5 | 29.4 | 18.6 |
| Mothers of U5 slept under LLIN or ITN | 52.5 | 54.8 | 25.2 | 25.5 | 38.7 | 40.1 | 28.4 | 29.8 | 9.8 | 28.5 | 37.2 | 44.5 | 23.8 | 36.4 | 7.0 | 12.8 | 32.4 | 60.4 | 31.2 | 38.7 |
| Child Vitamin A supplementation | 52.9 | 3.5 | 22.4 | 8.1 | 14.3 | 4.6 | 22.2 | 3.3 | 20.7 | 2.8 | 34.6 | 4.3 | 24.6 | 0.7 | 44.2 | 9.7 | 54.1 | 15.2 | 30.5 | 4.9 |
| U5 fever treatment with appropriate anti-malarial | 20.8 | 50.5 | 20.2 | 18.4 | 15.9 | 20.0 | 14.3 | 21.5 | 9.3 | 25.9 | 11.3 | 21.6 | 4.8 | 18.2 | 7.9 | 18.2 | 36.4 | 25.0 | 15.6 | 25.6 |
| U5 diarrhoea treatment with ORS | 44.3 | 60.3 | 32.4 | 40.8 | 34.5 | 56.6 | 29.3 | 49.5 | 47.7 | 63.7 | 30.6 | 49.2 | 28.9 | 38.0 | 40.6 | 37.6 | 58.4 | 60.0 | 36.0 | 51.5 |
| U5 ARI treatment for with appropriate antibiotics | 37.7 | 56.0 | 40.7 | 41.7 | 43.9 | 50.9 | 50.7 | 51.3 | 39.2 | 43.0 | 34.1 | 42.3 | 23.8 | 33.8 | 41.6 | 28.6 | 49.5 | 37.1 | 38.5 | 44.1 |
| **State average for all indicators** | 33.6 | 44.2 | 18.0 | 22.7 | 18.3 | 23.7 | 17.3 | 25.2 | 17.7 | 27.7 | 21.5 | 26.5 | 15.0 | 19.1 | 26.5 | 28.5 | 31.4 | 34.0 | 21.4 | 28.0 |

MNCH: Maternal, new-born and child health; ANC: Antenatal care; IPT2: Intermittent prevention therapy second dose; DPT3: Diphtheria, pertussis and tetanus vaccine; LLIN: Long lasting insecticide-treated bednets; ITN: Insecticide treated bednets; U5: Under five; ORS: Oral rehydration solution; Green coloured cells reached 50% coverage either in both 2011 and 2015 or in 2015 only

50% coverage goal achieved 40% coverage goal achieved 30% coverage goal achieved

**Supplementary Table S2. Significance tests for changes in coverage showing percentage change (as a decimal) and p-values for difference tests**

| **States** | **Central Equatoria** | **Eastern Equatoria** | **Jonglei** | **Lakes** | **Northern Bahr el Ghazal** | **Upper Nile** | **Warrap** | **Western Bahr el Ghazal** | **Western Equatoria** | **National** |
| --- | --- | --- | --- | --- | --- | --- | --- | --- | --- | --- |
| Contraceptive prevalence | 0.7 (0.472) | -1.3 (0.224) | -3.3 (<0.05*) | -3.1 (>0.05*) | 0.0 (>0.05*) | -1.7 (0.140) | -1.3 (>0.05*) | -7.9 (>0.05*) | -3.3 (0.187) | -1.5 (0.051) |
| 4+ ANC visits | -0.4 (0.425) | 7.5 (0.048) | 6.5 (0.018) | 3.2 (0.268) | 2.9 (0.386) | 5.0 (0.125) | -1.8 (0.248) | 1.8 (0.460) | -9.8 (0.016) | 2.6 (0.055) |
| 2+ Tetanus Toxoid vaccination | 43.1 (<0.001) | 32.4 (<0.001) | 35.6 (<0.001) | 50.0 (<0.001) | 49.4 (<0.001) | 30.0 (<0.001) | 34.6 (<0.001) | 39.8 (<0.001) | 42.8 (<0.001) | 39.7 (<0.001) |
| 2+ maternal IPT2 doses | 5.1 (0.264) | 9.6 (0.020) | 13.5 (0.002) | 14.0 (0.003) | 7.7 (0.174) | 18.4 (<0.001) | -1.7 (0.319) | 1.0 (0.492) | 5.0 (0.179) | 9.2 (<0.001) |
| Skilled birth attendance | 12.2 (0.046) | 3.6 (0.251) | 6.4 (0.034) | 15.4 (<0.001) | 5.4 (0.227) | -5.8 (0.081) | 0.0 (0.433) | 4.2 (0.334) | 8.4 (0.058) | 5.4 (0.001) |
| 1+ postnatal check-up | 10.0 (0.036) | 8.8 (0.013) | 6.6 (0.007) | -2.4 (0.161) | 19.8 (<0.001) | -3.7 (0.113) | 5.5 (0.092) | -12.8 (0.001) | 25.7 (<0.001) | 7.7 (<0.001) |
| DPT3 vaccination | 33.8 (<0.001) | 19.8 (<0.001) | 0.7 (0.468) | 12.2 (0.002) | 9.7 (0.036) | 3.8 (0.203) | 8.2 (0.043) | 15.2 (0.013) | 12.9 (0.003) | 14.3 (<0.001) |
| Full vaccination | 32.4 (<0.001) | 11.6 (0.003) | 0.2 (0.468) | 15.3 (<0.001) | 4.8 (0.166) | 13.0 (<0.001) | 5.5 (0.022) | 28.7 (<0.001) | 11.5 (<0.001) | 12.8 (<0.001) |
| U5 slept under LLIN | -4.8 (0.206) | -19.2 (<0.001) | -14.5 (0.002) | -4.1 (0.187) | 2.7 (0.371) | -3.1 (0.242) | -13.0 (0.004) | -8.5 (<0.05*) | -24.1 (<0.001) | -10.8 (<0.001) |
| Mother of U5 slept under LLIN | 2.3 (0.417) | 0.3 (0.476) | 1.4 (0.436) | 1.4 (0.444) | 18.7 (0.001) | 7.3 (0.085) | 12.6 (0.024) | 5.8 (0.140) | 28.0 (<0.001) | 7.5 (<0.001) |
| Child vitamin A supplementation | -49.4 (<0.001) | -14.3 (0.003) | -9.7 (0.001) | -18.9 (<0.001) | -17.9 (0.001) | -30.3 (<0.001) | -23.9 (<0.001) | -34.5 (<0.001) | -38.9 (<0.001) | -25.6 (<0.001) |
| U5 fever treatment with appropriate anti-malarial | 29.7 (<0.001) | -1.8 (0.295) | 4.1 (0.203) | 7.2 (0.069) | 16.6 (0.003) | 10.3 (0.005) | 13.4 (0.001) | 10.3 (0.031) | -11.4 (0.007) | 10.0 (<0.001) |
| U5 diarrhoea treatment with ORS | 16.0 (0.011) | 8.4 (0.081) | 22.1 (<0.001) | 20.2 (<0.001) | 16.0 (0.020) | 18.6 (<0.001) | 9.1 (0.084) | -3.0 (0.281) | 1.6 (0.421) | 15.5 (<0.001) |
| U5 ARI treatment with appropriate antibiotics | 18.3 (0.005) | 1.0 (0.468) | 7.0 (0.104) | 0.6 (0.500) | 3.8 (0.330) | 8.2 (0.046) | 10.0 (0.047) | -13.0 (0.016) | -12.4 (0.003) | 5.6 (0.001) |

*Used Yates-corrected chi square test, otherwise two-tailed two sample test for binomial proportions with normal approximation; ANC: Antenatal care; IPT2: Intermittent prevention therapy second dose; DPT3: Diphtheria, pertussis and tetanus vaccine; U5: Under five; LLIN: Long lasting insecticide-treated bednets; ITN: Insecticide treated bednets; ORS: Oral rehydration solution; Green coloured cells reached 50% coverage either in both 2011 and 2015 or in 2015 only

Statistically significant improvement Statistically significant decline Non-statistically significant change
